# Single-Camera Trap Survey Designs Miss Detections: Impacts on Estimates of Occupancy and Community Metrics

**DOI:** 10.1371/journal.pone.0166689

**Published:** 2016-11-30

**Authors:** Brent S. Pease, Clayton K. Nielsen, Eric J. Holzmueller

**Affiliations:** 1 Department of Forestry, Southern Illinois University, Carbondale, IL, United States of America; 2 Cooperative Wildlife Research Laboratory, Southern Illinois University, Carbondale, IL, United States of America; University of Kwazulu-Natal, SOUTH AFRICA

## Abstract

The use of camera traps as a tool for studying wildlife populations is commonplace. However, few have considered how the number of detections of wildlife differ depending upon the number of camera traps placed at cameras-sites, and how this impacts estimates of occupancy and community composition. During December 2015–February 2016, we deployed four camera traps per camera-site, separated into treatment groups of one, two, and four camera traps, in southern Illinois to compare whether estimates of wildlife community metrics and occupancy probabilities differed among survey methods. The overall number of species detected per camera-site was greatest with the four-camera survey method (P<0.0184). The four-camera survey method detected 1.25 additional species per camera-site than the one-camera survey method, and was the only survey method to completely detect the ground-dwelling silvicolous community. The four-camera survey method recorded individual species at 3.57 additional camera-sites (P = 0.003) and nearly doubled the number of camera-sites where white-tailed deer (*Odocoileus virginianus*) were detected compared to one- and two-camera survey methods. We also compared occupancy rates estimated by survey methods; as the number of cameras deployed per camera-site increased, occupancy estimates were closer to naïve estimates, detection probabilities increased, and standard errors of detection probabilities decreased. Additionally, each survey method resulted in differing top-ranked, species-specific occupancy models when habitat covariates were included. Underestimates of occurrence and misrepresented community metrics can have significant impacts on species of conservation concern, particularly in areas where habitat manipulation is likely. Having multiple camera traps per site revealed significant shortcomings with the common one-camera trap survey method. While we realize survey design is often constrained logistically, we suggest increasing effort to at least two camera traps facing opposite directions per camera-site in habitat association studies, and to utilize camera-trap arrays when restricted by equipment availability.

## Introduction

The use of remotely-triggered cameras (hereafter, camera traps) as a tool for studying wildlife populations is commonplace [1, 2]. Recent areas of research utilizing camera traps include studies of species distribution [3], community dynamics [4], population densities [5], and occupancy modeling [6–7]. Noteworthy increases in camera trap deployment [8], however, have prompted investigation into camera trap survey design to address the sometimes low detection rates associated with this survey method [9]. Deploying attractants at a camera-site has been used to increase detection rates [10], although this technique can affect which species or functional groups are detected at a camera-site [2]. Others have implemented non-random camera trap placement to increase detections, but this design can violate key assumptions of random sampling [6]. Further, research indicates even minor adjustments to camera trap placement at a determined sampling location can influence which species are detected during surveys [8, 11–13].

Camera trap surveys are also constrained logistically due to costs associated with field equipment, often limiting the total number of cameras available to one per site to maximize the number of sites sampled [14]. In the common situation of limited resources, and as species of conservation concern are often the focal point in camera trapping, identifying designs that maximize efficiency (camera trap placement) and accuracy (detection rates) is a crucial step to refining camera trapping studies [15]. Indeed, missed detections as a result of resource-limited survey designs have been shown to have greater consequences on parameter estimations than measurement error in habitat covariates associated with a sampling unit, where additional sampling effort has been encouraged to reduce bias and improve model predictions [16].

Across wildlife survey techniques, and particularly in presence/absence surveys utilizing camera traps, researchers experience false-negative measurement errors when a species is recorded as absent despite the species’ true presence [16–19]. Additional efforts to reduce this measurement error have included deploying multiple detection devices (e.g. one camera trap and one track-plate) at the same sampling unit [20] and arranging camera traps in unique spatial patterns to increase detection rates (e.g. [21]). While these efforts did improve detection rates, few studies have considered increasing the number of camera traps deployed per camera-site as an attempt to reduce this measurement error [20, 22]. Rather, a common survey design entails deploying one camera trap per camera-site aimed in the direction researchers predict to have the highest probability of detection for the target species, and widely distributing the camera-sites across an area of interest [11, 23, 24]. In this design, the likelihood of experiencing a false-negative error seems inherent as, depending on the camera model used, nearly 90% of the individual camera-site is not assessed due to the limited field-of-view of a fixed remote camera [25]. Nonetheless, while it is assumed that a more robust wildlife survey method would improve the detection rate of a species, thus reducing the false-negative error rate, relatively few attempts to evaluate design efficacy utilizing camera traps have been made [20]. Given the challenges associated with camera trap survey design, coupled with imperfect detection of many terrestrial mammals [17, 26], further investigation into camera trap placement to better understand how to maximize detection rates and thus more accurately calculate community metrics is warranted.

The goal of our study was to investigate how detection rates represented by one, two, and four camera traps per camera-site would affect estimates of occupancy and other wildlife community metrics. We hypothesized that the deployment of one camera trap per camera-site would incompletely record the terrestrial mammal species utilizing the camera-site. We predicted that by increasing the number of camera traps per camera-site we would significantly increase the number of species detected. This increase will result in different occupancy modeling results and potentially reveal habitat relationships not detected by encounter histories generated from one-camera surveys.

## Methods

### Ethics statement

Data collection used non-invasive, remotely-triggered camera traps and hence did not involve direct contact or interaction with the animals. No bait or lure was used to further limit interference with animals. Fieldwork was done under research permit number SS15-42 to EJH, issued by the Illinois Department of Natural Resources (IDNR).

### Study area

**Fig 1:**
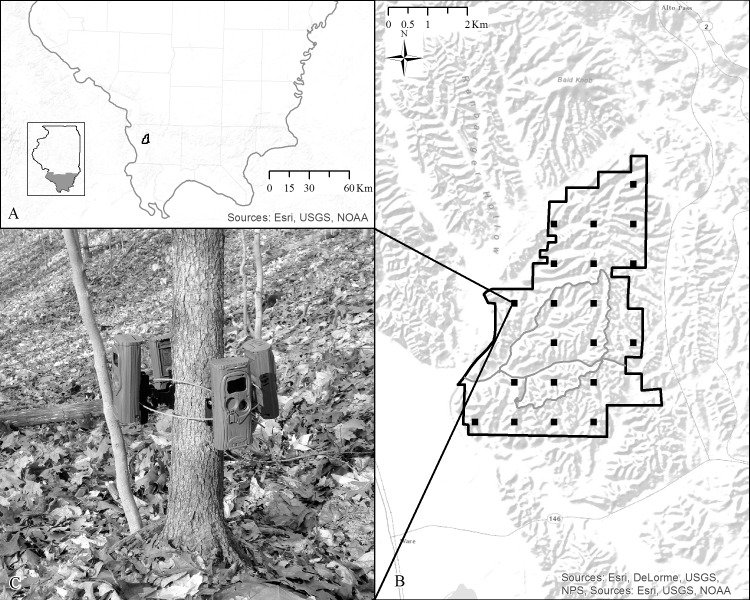
Location of Trail of Tears State Forest in southwestern Illinois, USA. Black squares represent camera-site locations. Panel (A) shows the location of the study site within the state of Illinois, USA, panel (B) illustrates the distribution of camera-sites at the study site, and panel (C) depicts how camera traps were deployed and arranged at a camera-site (Basemap source: ESRI, Redlands, CA, USA).

Trail of Tears State Forest (37 22’ N, 89 22’ W; TTSF) is situated west of the neighboring Mississippi River Floodplain in Union County, Illinois (Fig 1A). Consisting of 2088 ha, TTSF is in the easternmost section of the Ozark Plateau and is one of the largest blocks of contiguous forest in the lower Midwest [27]. The topography is heavily eroded and primarily made up of long, narrow forested ridge tops, steeply leading (15–44% slopes) to ravine bottoms 45–60 m below [28]. Elevation ranges from 106 m– 320 m above sea level [28], and the overstory forest cover is a mosaic of mature oak-hickory (*Quercus spp*.*–Carya spp*.) with midstory and understory components of Sugar Maple (*Acer saccharium*) and American Beech (*Fagus grandifolia*) on upland sites, while mature mixed hardwoods exist at lower elevations including a significant component of Yellow Poplar (*Liriodendron tulipifera*) [27, 29]. Average high and low temperatures are 18°C and 7°C, respectively, with an annual precipitation of 128 cm [30].

### Camera trap surveys

Camera trapping was conducted during December 2015─ February 2016. Using function ‘create a fishnet’ in ArcGIS 10.3 [31], we created a systematic sampling grid in the political boundary of TTSF with cell sizes of 1 km^2^ and generated sampling points at the center of each cell, resulting in 23 camera-sites with a distance of one km between each site (Fig 1B). Three site were removed because they were placed in human development and outside of forested habitat, reducing the total number of sampled camera-sites to 20. At each camera-site we deployed four digital remote camera traps (Cuddeback E2 [20.0 megapixel], Attack IR [5.0 megapixel], or Ambush IR [5.0 megapixel], Non Typical, Inc., Park Falls, WI) which were furnished with passive infrared sensors that trigger the camera when a rapid change is detected in the surface temperature of an object [32], and were equipped with incandescent flash illumination to assist in species identification at night. Additionally, all camera traps used were furnished with one-fourth second trigger speeds and were capable of a flash range of at least 30 m. The four cameras were deployed on the same tree bole with even spacing among the cameras so as to have ~90° spacing between cameras (Fig 1C). We selected a tree within 10 m of our generated sampling point that provided a field of view for the survey and, when available, allowed for one camera to be directed at a wildlife trail. On the same tree, the remaining three cameras were mounted so that all four cameras were deployed at a height of 40–50 cm above ground level [33]. Cameras were deployed for 30 days and were set to be active 24-hours each day with 30-second delays between photographs; cameras recorded one image per trigger and each photo recorded the date and time of the event, and we identified species present in each photo. We used a threshold of 60 minutes to temporally distinguish independence of unique photographic events of the same species [11]. Camera deployment was passive in that no bait or lure was used at the camera-sites [2]. When the number of camera traps available is a limiting factor, camera trap arrays, or sets of camera traps, can be deployed sequentially rather than simultaneously [2, 33]. Given this, camera traps were deployed in two arrays of 10 camera-sites [33].

### Camera data preparation

Camera traps at a camera-site were grouped into ‘treatments’ to compare detection rates of one-, two-, and four-camera survey methods: (1) one camera trap facing nearby signs of wildlife (herein, one-camera survey method), (2) a pair of camera traps on opposite sides of the tree (herein, two-camera survey method), and (4) the overall group of four camera traps (herein, four-camera survey method). The two-camera survey method included the camera trap used in the one-camera survey method and a camera trap located on the opposite side of the same tree. The four-camera survey method included all of the camera traps at a camera-site–the two-camera survey method with two additional cameras. These ‘treatments’ were nested in time and space so that we could directly quantify the number of in-situ missed detections. Survey occasions were defined as five days, resulting in five unique surveys at each camera-site. We calculated survey effort as the number of calendar days a camera trap was operational multiplied by the number of operational camera traps because our methodology was designed so that each camera trap at a camera-site collected data and accumulated effort independently from the other cameras present at that particular camera-site. This accumulation of survey effort is to contrast with studies of spatial capture-recapture which deploy 2 opposing cameras to capture each flank of the individual. The paired camera traps are collecting the same data and therefore resulting in one camera day of effort rather than two, despite the deployment of two cameras per camera-site [24].

### Habitat characteristics

We collected forest overstory and understory metrics at each camera-site to parameterize species-specific occupancy models. Forest characteristics often collected during habitat use studies (e.g. canopy cover, ground cover) were not available given the winter season, thus we relied on topographic characteristics and forest structure measurements [34]. At each camera location we measured all woody overstory stems ≥ 5 cm diameter at breast height (dbh) within a variable-radius plot (10-factor prism) to determine density and basal area. We also tallied all woody understory, < 5 cm dbh and ≥ 1.37 m tall, within a 3.6 m fixed-radius plot centered at cameras to estimate understory stem density by species. Using ArcGIS 10.3 [31], we measured elevation, percent slope, and aspect of each camera-site using a 30 arc-second digital elevation model (DEM). We also used precipitation and temperature data recorded at the nearest weather station for use in model building [4, 30].

### Wildlife community dynamics

We compared wildlife community metrics represented by the one-, two-, and four-camera survey methods with single-factor analysis of variance (ANOVA) using program R’s built-in ANOVA functions [35]. ANOVA was used to determine if the number of cameras deployed at a camera-site had an effect on the number of detections and species richness. Significant models were further analyzed with Tukey’s HSD test to determine differences among treatment means. All tests carried out were evaluated at the alpha = 0.05 level. To meet ANOVA’s basic assumptions of normality and homoscedasticity, data were log_10_ transformed and normal quantile plots (Q-Q Plots) were evaluated.

### Occupancy modeling

Introduced in MacKenzie *et al*.'s (2002) seminal paper, occupancy modeling is a hierarchical framework developed to account for the measurement error associated with the imperfect detection of a species through a series of repeated surveys at multiple locations within a defined season, where the target species is either detected with probability *p*, or not detected (1-*p*). The detection probability parameter (*p)*, the probability of detecting a species given it is present, accounts for the false-negative measurement error, which is the compliment of detection probability (1-*p)*. When a species is detected during a visit, *j*, the visit is assigned a value of “1” and when non-detection occurs, it is denoted with a “0”; a matrix of 1s and 0s is developed from multiple visits and sites to determine a species’ encounter history. This encounter history matrix is ultimately used to evaluate the state parameter of interest, occupancy, Ψ, or the probability that a species is present at site *i* [6, 36, 37].

We generated species-specific detection histories from surveys and developed models using program R’s package, ‘*unmarked’* [35, 38]. Package ‘*unmarked’* fits hierarchical models to imperfectly detected species occurrence and abundance datasets [38]. While the package offers modeling for advanced, dynamic designs, we utilized *unmarked* for static, single-season site-occupancy models developed by Mackenzie *et al*. (2002) to estimate the detection process and the probability of site occupancy. We used the link function during model building to express the effects of environmental covariates that varied spatially and temporally. Covariates included averages of precipitation and temperature during each survey period and survey-period specific intercepts, as well as amount of hardwood basal area (m^2^ ha^-1^), distance to forest edge, and sapling abundance per site [4]. Additionally, topographic characteristics including percent slope, site elevation, and forest aspect were offered to models. We used a step-wise process to identify the model parameters that best explained our data, which entailed initially holding Ψ constant (null model Ψ(.)) while fitting the measurement error model of detection probability. Once the top detection model was identified we then fitted a suite of occupancy models to habitat covariates collected that could explain the distribution of the terrestrial wildlife community using our study site. Models were ranked according to their Akaike’s Information Criterion (AIC) value and model weight, and we considered those ≤ 2 AIC points of top model as competitive models [39]. We omitted occupancy analysis of eastern gray squirrels (*Sciurus sciurus*) and raccoons (*Procyon lotor*) as they occurred at almost all camera-sites, and eastern-wild turkeys (*Meteagris gallopava silvestris*) because of their overall low detection rates during our surveys [4].

We fitted derived detection histories by one-, two-, and four-camera survey methods to develop baseline null models as well as models which incorporated measured habitat covariates, and to identify whether model selection varied with survey effort. For each focal species, we fitted a null model [Ψ (.)p(.)] under one-, two-, and four-camera detection histories to develop a working baseline. From there, we identified the best approximating model given the data for all survey methods. We used the compliment of the estimated detection probability from the null models to estimate the species-specific measurement error under each camera survey method.

## Results

### Detections

We recorded 2386 camera-days and 688 photographs of target species with the four-camera survey method at 20 camera-sites (Table 1). Among those photographs, 34% were eastern gray squirrels, 29% white-tailed deer (*Odocoileus virginianus*), 27% raccoons, 4% coyotes (*Canis latrans*), 3% Virginia opossums (*Didelphis Virginiana*), 2% bobcats (*Lynx rufus*), and 2% eastern wild turkeys. The two-camera method resulted in 369 photographs from 1190 camera-days of effort, and the one-camera survey method yielded 200 photographs with an effort of 592 camera-days (Table 1).

**Table 1:** Species detected in southern Illinois during Dec 2015 –Feb 2016, and the number of detections recorded and retained for analysis.

Species	One Camera	Two Cameras	Four Cameras
Bobcat	5	7	10
Coyote	5	6	13
Eastern Gray Squirrel	57	75	124
Eastern Wild Turkey	2	4	5
Virginia Opossum	9	10	15
Raccoon	29	61	102
White-tail deer	17	27	40
Total recorded	200	369	688
Analysis total^a^	124	190	309
Number of camera days	592	1190	2386
Total photographs/camera-day	0.338	0.310	0.288

The total numbers of photographs recorded for each species (Total recorded) and the total number of photographs used in analyses (Analysis total) are given for each survey method.

^a^Total number of photographs (detections) used in data analysis for each species after removing photographs taken within 60 minutes of another photo at the same camera location.

### Wildlife community metrics

**Fig 2:**
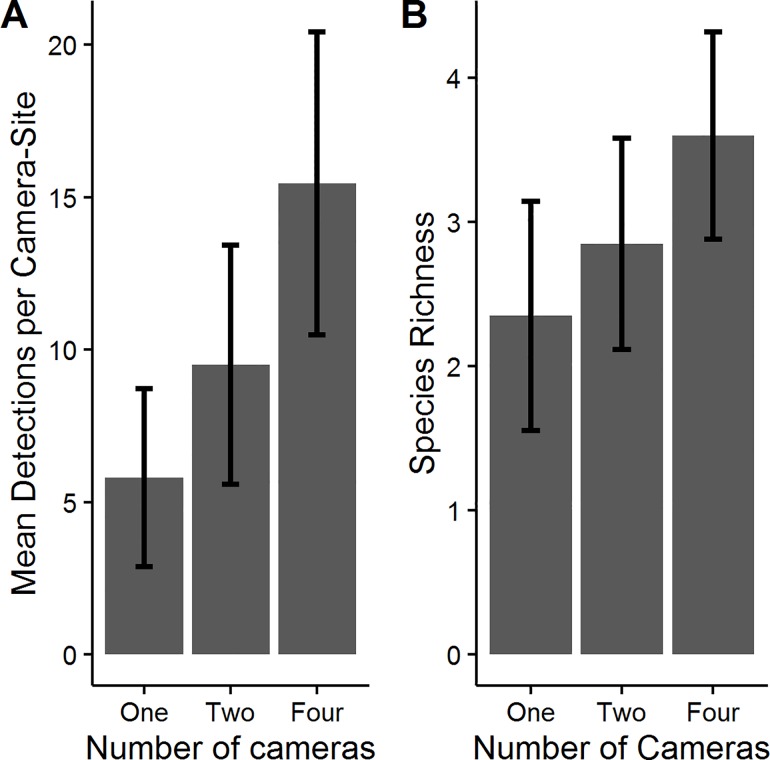
Comparison of (A) mean detections and (B) species richness detected per camera-site represented by one-, two-, and four-camera camera survey methods.

The number of detections recorded differed among the camera survey methods (F_2,57_ = 7.8515, P = 0.0009; Fig 2A). Results from Tukey’s HSD test indicate detection rates differed between one- and four-camera survey methods (P<0.05), with no other differences significant. Overall, we saw a 64% increase in mean detections from one to two cameras per camera-site, and a 63% increase from two to four cameras per camera-site (Table 2). There was a notable difference in the number of species detected per camera survey method (F_2,57_ = 4.28, P = 0.019), with significant differences among only one- and four-camera survey methods (P<0.05). The four-camera survey method detected 1.25 (53%) additional species than the one-camera survey method and 0.75 (26%) additional species than the two-camera survey method (Fig 2B; Table 2). The four-camera survey method was the only method to completely detect the ground-dwelling silvicolous community (bobcat, coyote, eastern gray squirrel, eastern wild turkey, Virginia opossum, raccoon, white-tailed deer) utilizing TTSF at a single camera-site (n = 7 species).

**Table 2:** Detection events and species richness made by one-, two-, and four-camera survey methods in southern Illinois, Dec 2015 –Feb 2016.

	One Camera		Two Cameras		Four Cameras			
	$\bar{x}$	SE	$\bar{x}$	SE	$\bar{x}$	SE	F-value	Pr>F^a^
Detections	5.8	1.4	9.5	1.87	15.45	2.37	7.85	0.0009
Species Richness	2.35	0.38	2.85	0.35	3.6	0.34	4.28	0.0184

^a^Tests were carried out at the alpha = 0.05 level.

The one-camera survey method detected focal species at an average of 6.7 sites (range 2–13) while four-camera surveys resulted in detections at 10.3 sites (range 3–19; Table 3). The four-camera survey method increased detections across all taxonomic and functional groups, where the one-camera survey method missed the region’s most common mammalian species detectable using camera traps (white-tailed deer, raccoon, eastern gray squirrel) at an average of 5.3 sites (Table 3). The number of sites where a species was detected among the one- and two-camera survey methods differed by 21% (Table 3).

**Table 3:** Number of sites at which each species was detected in southern Illinois during Dec 2015-Feb 2016 for one-, two- and four-camera survey methods.

Species	One Camera	Two Cameras	Four Cameras
Bobcat	3	3	5
Coyote	3	3	5
Eastern Gray Squirrel	13	14	17
Eastern Wild Turkey	2	3	3
Virginia Opossum	5	6	9
Raccoon	13	16	19
White-tailed Deer	8	12	14
$\bar{x}$	6.71	8.14	10.29

### Occupancy modeling

Detection history matrices differed among each survey method, resulting in different per-species estimates of occupancy and detection probability across survey methods (Table 4). At times, detection histories were identical, thus estimates did not differ between survey methods (e.g. one- and two-camera survey methods for coyotes; Table 4). In general, four-camera methods increased detection probabilities with a decreasing standard error, and occupancy estimates were closer to that of the naïve estimates (Table 4). For example, four-camera survey methods identified white-tailed deer at 70% of the camera-sites, and the generated occupancy estimate was nearly identical (Ψ = 0.718, Table 4). Detection probabilities increased as the number of cameras increased at a camera-site for all species except Virginia opossums, where a negative relationship existed (Table 4). Occupancy modeling with site-specific habitat covariates resulted in differing top models among one- and four-camera survey methods for all species, and the top models for one- and two-camera survey methods differed for all species except coyotes (AIC values; Table 5).

**Table 4:** Estimates of state parameters in occupancy modeling derived from detection histories gathered in southern Illinois, Dec 2015 –Feb 2016.

Species	Method	Naïve Ψ ^a^	Ψ(.)^b^	*p* (.)^c^	SE(*p*)^d^	Measurement Error (1-*p)*
Bobcat	One Camera	0.15	1.000	0.030	0.017	0.970
	Two Cameras	0.15	0.277	0.144	0.125	0.856
	Four Cameras	0.25	0.414	0.169	0.101	0.831
Coyote	One Camera	0.15	0.277	0.144	0.125	0.856
	Two Cameras	0.15	0.277	0.144	0.125	0.856
	Four Cameras	0.25	0.414	0.169	0.101	0.831
Virginia Opossum	One Camera	0.25	0.294	0.204	0.118	0.796
	Two Cameras	0.30	0.414	0.169	0.101	0.831
	Four Cameras	0.45	0.894	0.113	0.071	0.887
White-tailed Deer	One Camera	0.40	0.557	0.179	0.087	0.821
	Two Cameras	0.60	0.685	0.277	0.074	0.723
	Four Cameras	0.70	0.718	0.376	0.069	0.624

Naïve occupancy estimates were calculated by methods presented in MacKenzie *et al*. (2002), and represent the proportion of total sites at which a species was detected. Occupancy and detection estimates presented are the transformed beta estimates from the null model [Ψ(.) *p*(.)].

^a^The proportion of sites a species was actually detected

^b^Occupancy probability–the estimation of the proportion of sites occupied, given the detection history of a species.

^c^Detection probability–the probability of detecting a species, given it is present.

^d^Standard Error of detection probability estimates.

**Table 5:** Comparison of the top fitting and null models in occupancy modeling under each survey method for species detected at southern Illinois, Dec 2015 –Feb 2016.

Species	Method	Model^a^	K^b^	AIC^c^	ΔAIC^d^	ω^e^
Bobcat	One Camera	Ψ (EDGE + ELEVATION) *p* (FULL + PRECIP)	6	17.63	0.00	0.618
		NULL^f^	2	30.95	13.32	0.001
	Two Cameras	Ψ (EDGE + ELEVATION) *p* (FULL X PRECIP)	5	24.28	0.00	0.869
		NULL	2	36.14	11.86	0.002
	Four Cameras	Ψ (.) *p* (FULL X PRECIP)	3	48.92	0.00	0.147
		NULL	2	53.00	4.08	0.019
Coyote	One Camera	Ψ (UNDERSTORY + ELEVATION) *p* (TEMP X PRECIP)	5	30.05	0.00	0.195
		NULL	2	36.14	6.09	0.009
	Two Cameras	Ψ (UNDERSTORY + ELEVATION) *p* (TEMP X PRECIP)	5	30.05	0.00	0.195
		NULL	2	36.14	6.09	0.009
	Four Cameras	Ψ (BA + ELEVATION) *p* (.)	4	48.51	0.00	0.281
		NULL	2	53.00	4.49	0.029
Virginia Opossum	One Camera	Ψ (EDGE + SLOPE) *p* (PRECIP + TEMP)	6	26.92	0.00	0.760
		NULL	2	46.40	19.18	0.000
	Two Cameras	Ψ (ELEVATION) *p* (PRECIP + TEMP)	5	45.15	0.00	0.126
		NULL	2	53.00	7.85	0.002
	Four Cameras	Ψ (EDGE) *p* (PRECIP + TEMP)	5	54.74	0.00	0.135
		NULL	2	68.98	14.24	0.000
White-tailed Deer	One Camera	Ψ (BA + EDGE) *p* (.)	4	58.25	0.00	0.552
		NULL	2	67.63	9.38	0.005
	Two Cameras	Ψ (EDGE + SLOPE) *p* (.)	4	82.99	0.00	0.960
		NULL	2	98.82	15.83	0.000
	Four Cameras	Ψ (SLOPE + ELEVATION) *p* (FULL X PRECIP)	5	110.53	0.00	0.326
		NULL	2	115.98	5.45	0.021

Models were built in the statistical software R package ‘unmarked’[38]. Each survey method was offered identical detection and occupancy covariates. The top-ranked model for each species-specific survey method is given followed by the null model.

^a^ BA, amount of basal area per camera-site; EDGE, distance to TTSF boundary; ELEVATION, meters above sea level at camera-site; FULL, unique detection probability per survey event; PRECIP, sum of precipitation recorded during survey period; SLOPE, the landscape grade of the camera-site; TEMP, average temperature recorded during survey period; UNDERSTORY, number of woody stems taller than 1.37 m and less than 5 cm dbh; (.), fixed, constant parameter among camera-sites or surveys.

^b^Number of model parameters

^c^Akaike's Information Criterion

^d^Change in AIC value from the top-ranked model.

^e^Model weight; the probability of a model being the best approximating model among those evaluated.

^f^Ψ(.)*p*(.)

## Discussion

We quantified how detection rates and species detected would differ when treatment groups of one, two, and four camera traps were deployed at a camera-site, and how those changes would affect occupancy modeling outcomes and wildlife community metrics. We observed increases in detection rates, the number of sites where a species was detected, and per camera-site species richness as we increased the number of cameras at a camera-site. Additionally, the increased detection rates derived from the four-camera survey method revealed habitat relationships not detected by one- or two-camera survey efforts in occupancy modeling. Our findings have implications for general camera trap survey design and resource allocation, and particularly for researchers utilizing camera traps in studies of occupancy.

For the one-camera survey method, we aimed the single camera trap at nearby animal-made trails when they were present, as literature suggests detection probabilities can be higher for several mammal species, particularly carnivores, with this directional deployment [11, 40]. However, we found that increasing the number of cameras at a camera-site aimed away from game trails resulted in a higher proportion of sites where carnivores (e.g. bobcat, coyote) were detected relative to the one-camera survey method. Some have suggested increased detection rates with trail-based camera traps has geographical consistency [41], where game-trail use appears to be relatively high in Central and South America. Patterns of trail-use has also been credited to the width of game-trails, where wider trails provide a strong contrast with the surrounding vegetation, thus increasing trail use by wildlife [41]. To our knowledge, however, no other study has compared how detection rates vary with camera-trap placement and survey effort in a temperate ecosystem during the winter season. In this scenario, there is little to no vegetation creating understory structure in the forest, thereby creating a lack of contrast among trails and surrounding areas. Thus, the results we found may be due to seasonal movement patterns in our habitat type (i.e., the Central Hardwood Forest) [4,42], as animal movements may follow trails less frequently when leaves are off of the understory vegetation [40]. The lack of trail use could also be due to sparse prey availability during winter seasons, which can contribute to less predictable movement patterns in carnivores and larger home ranges [12]. Additionally, our surveys coincided with the breeding-gestation season of bobcats which could have influenced our non-targeted detection rates, where increased random movements of males while searching for females can be observed [42].These results may have implications for further research on carnivores utilizing camera traps in North America during their breeding-gestation season. Often camera surveys of carnivores occur during the breeding season [4]; in those cases, deploying more cameras randomly throughout the study area rather than concentrating survey efforts to trails may result in increased detection rates.

While increasing sampling effort to two- and four-cameras per camera-site did indeed increase detection rates, one-camera survey methods recorded the same overall number of species as the other combination of camera traps, with significantly fewer calculated camera-days. This concurs with previous literature that showed one randomly distributed camera trap directed towards wildlife trails adequately detected ground-dwelling communities [33]. However, questions of habitat associations can be of more interest than inventories, and our results indicate per-unit sampling effort can greatly affect relationships detected when using occupancy modeling. Gu and Swihart (2004) demonstrated the effect of false-negative errors on wildlife-habitat models, and extended the need for repeated surveys (temporal replication) across a number of sites (spatial replication). As MacKenzie and Royle (2005) discuss, however, increasing the number of sites in occupancy studies may not always result in the most precise state parameter estimates, suggesting that concentrating survey effort on fewer sites can reduce statistical and measurement error. Our results demonstrate this, showing that researchers would experience a high rate of measurement error when reducing the number of camera traps per site but increasing the number of sites surveyed, thus potentially misguiding habitat associations detected during occupancy modeling. Further, our results show that no species-specific detection history generated the same best-fitting habitat model between the one- and four-camera survey methods, suggesting directional placement and number of camera traps at the camera-site can greatly influence detection probabilities of individual species, which again affects the relationships discovered [17].

Across several species, we saw unanticipated baseline (null) occupancy and detection estimates (one-camera: bobcat, four-camera: Virginia opossum), where low detection rates across all sites resulted in occupancy estimates of nearly 1. Again, this issue arises when the number of detections across all sites are always low, resulting in maximum likelihood estimates of a very high (and unlikely) occupancy rate with an extremely low detection probability [43]. This can be of particular importance for rare and cryptic species, which often generate detection histories dominated by 0s (absences). False-zero dominated matrices are expected for rare species, but we found that four-camera survey methods can reduce this estimation error. Even if a species is detected at an identical number of sites across survey methods, the sequence and frequency of detection events at those camera-sites can impact model building. For example, the Virginia opossum detection history compiled by four-camera survey methods, though consisting of more ‘present’ events, resulted in unrealistic null estimates of state parameters (e.g. occupancy probability = 1). This is likely due to the low number of camera-sites and is a common issue in conservation-related projects [43].

Camera trap survey design and efforts are ultimately constrained logistically, whether that is by limited resources or difficult field conditions. Thus, researchers will likely be faced with the trade-off of decreasing the per-unit sampling effort but increasing the number of sites surveyed, or increasing survey intensity over a smaller area with fewer camera-sites. Several authors have provided survey design suggestions [15, 19, 43], which can guide a researcher’s allocation of effort, however some of the provided guidelines require knowledge of expected state parameter estimates which may not always be available. Like others (e.g. [44]), we suggest camera trap survey design is dependent on the focal research questions. In habitat-use research whose ecological scale is 3^rd^ order [45] or smaller, we recommend a minimum of 2 camera traps per camera-site, deployed towards opposite directions to increase detection rates. We acknowledge that it is unlikely research efforts will be able to quadruple their survey effort (e.g. four-camera survey methods), as this was carried out in our study merely to demonstrate the number of missed detections. However, by simply doubling per-unit effort an insignificant number of detections and species are missed, thereby aiding the occupancy model building process and addressing the sometimes low detection rates associated with camera trapping studies.

## Supporting Information

S1 Dataset Camera trap survey dataset used for analysis in this study. (CSV)
